# Repositioning of the β-Blocker Carvedilol as a Novel Autophagy Inducer That Inhibits the NLRP3 Inflammasome

**DOI:** 10.3389/fimmu.2018.01920

**Published:** 2018-08-22

**Authors:** Wei-Ting Wong, Lan-Hui Li, Yerra Koteswara Rao, Shih-Ping Yang, Shu-Meng Cheng, Wen-Yu Lin, Cheng-Chung Cheng, Ann Chen, Kuo-Feng Hua

**Affiliations:** ^1^National Defense Medical Center, Graduate Institute of Life Sciences, Taipei, Taiwan; ^2^Department of Laboratory Medicine, Linsen, Chinese Medicine and Kunming Branch, Taipei City Hospital, Taipei, Taiwan; ^3^Department of Biotechnology and Animal Science, National Ilan University, Yilan City, Taiwan; ^4^Division of Cardiology, Department of Internal Medicine, Tri-Service General Hospital, National Defense Medical Center, Taipei, Taiwan; ^5^Department of Pathology, Tri-Service General Hospital, National Defense Medical Center, Taipei, Taiwan; ^6^Department of Medical Research, China Medical University Hospital, Taichung, Taiwan

**Keywords:** Carvedilol, NLRP3 inflammasome, autophagy, mitochondria, peritonitis

## Abstract

The NLRP3 inflammasome is a multiprotein complex that plays a key role in the innate immune system, and aberrant activation of this complex is involved in the pathogenesis of inflammatory diseases. Carvedilol (CVL) is an α-, β-blocker used to treat high blood pressure and congestive heart failure; however, some benefits beyond decreased blood pressure were observed clinically, suggesting the potential anti-inflammatory activity of CVL. In this report, the inhibitory potential of CVL toward the NLRP3 inflammasome and the possible underlying molecular mechanisms were studied. Our results showed that CVL attenuated NLRP3 inflammasome activation and pyroptosis in mouse macrophages, without affecting activation of the AIM2, NLRC4 and non-canonical inflammasomes. Mechanistic analysis revealed that CVL prevented lysosomal and mitochondrial damage and reduced ASC oligomerization. Additionally, CVL caused autophagic induction through a Sirt1-dependent pathway, which inhibited the NLRP3 inflammasome. In the *in vivo* mouse model of NLRP3-associated peritonitis, oral administration of CVL reduced (1) peritoneal recruitment of neutrophils; (2) the levels of IL-1β, IL-18, active caspase-1, ASC, IL-6, TNF-α, MCP-1, and CXCL1 in the lavage fluids; and (3) the levels of NLRP3 and HO-1 in the peritoneal cells. Our results indicated that CVL is a novel autophagy inducer that inhibits the NLRP3 inflammasome and can be repositioned for ameliorating NLRP3-associated complications.

## Introduction

Carvedilol (CVL) belongs to a class of drugs called α-, β-blockers, which are generally used for cardiovascular disorders. CVL blocks sympathetic neural activation via antagonism of β1-, β2-, and α1-adrenoreceptors and has shown greater cardiovascular benefits than traditional β-blockers in both humans and animals. Several *in vivo* and *in vitro* studies have demonstrated the cardioprotective, nephroprotective and hepatoprotective effects of CVL against various toxicant-induced preclinical models, and these effects are independent of its β-adrenergic blocking properties (1). Versatile anti-inflammatory effects of CVL were observed in both *in vitro* and *in vivo* studies. For example, our previous studies showed that CVL inhibited T cell activation by suppressing NF-κB activity (2). CVL reduced interleukin (IL)-1β-mediated upregulation of matrix metalloproteinase in chondrocytes, indicating the chondroprotective potential of CVL (3). Additionally, CVL protected against cisplatin-induced renal toxicities in tumor-bearing mice (4). Furthermore, CVL suppressed the plasma levels of tumor necrosis factor (TNF)-α and IL-6 in both ischaemic and non-ischaemic patients (5). The anti-inflammatory effects of CVL may be associated with its reactive oxygen species (ROS)-scavenging effects (6). However, the effect of CVL on the NLRP3 inflammasome, the caspase-1-containing protein complex that controls IL-1β and IL-18 secretion, remains unclear.

NLRP3 inflammasome senses and can be activated in response to a highly diverse range of pathogens and environmental and endogenous danger molecules, such as SiO2, ATP, the pore-forming toxin nigericin, cholesterol crystals (CC), monosodium urate crystals (MSU), amyloid-β, and islet amyloid polypeptide (7). Although the NLRP3 inflammasome is important in innate immunity to fight infection, excessive activation of this complex is involved in a variety of common diseases, including gout, atherosclerosis, type 2 diabetes, neurodegenerative diseases, and cardiovascular disorders (8, 9). Therefore, NLRP3 inflammasome activity and the associated signaling pathways are common targets for next-generation therapeutics (10). The currently available clinical treatment for NLRP3-related diseases is agents that target IL-1β, including the recombinant IL-1 receptor antagonist anakinra, the IL-1β-neutralizing antibody canakinumab, and the soluble decoy IL-1β receptor rilonacept (11). Additionally, several compounds, including sulforaphane, isoliquiritigenin, β-hydroxybutyrate, flufenamic acid, mefenamic acid, 3,4-methylenedioxy-β-nitrostyrene, parthenolide, BAY 11-7082, INF39, and MCC950, have shown potent inhibition of the NLRP3 inflammasome (9, 12, 13). However, these strategies have a number of potential undesirable effects, which are frequently associated with the suppression of a major cytokine involved in many NLRP3-dependent and independent physiological processes. For example, sulforaphane is not specific to the NLRP3 inflammasome; it also inhibits the AIM2 or NLRC4 inflammasome and NF-κB activation (14). Thus, there is a need to identify novel small molecules targeting the NLRP3 inflammasome, which in general are more cost-effective than biological agents (15).

In the present study, we assessed the effect of CVL on the NLRP3 inflammasome *in vitro* and *in vivo*. The effects of CVL on the signaling pathways involved in the regulation of NLRP3 inflammasome activation were investigated, including lysosomal rupture, mitochondrial damage, ASC oligomerization, and autophagy induction. Additionally, we assessed the *in vivo* effects of CVL in a mouse model of NLRP3-associated peritonitis by analysing the peritoneal recruitment of neutrophils and the levels of cytokine in the lavage fluids. To the best of our knowledge, this is the first report describing the *in vitro* and *in vivo* beneficial effects of CVL on the NLRP3 inflammasome.

## Materials and methods

### Materials

CVL, lipopolysaccharide (LPS) (from *Escherichia coli* 0111:B4), colchicines, 3-methyladenine (3-MA), phorbol myristate acetate, N-acetylcysteine (NAC), monodansylcadaverine (MDC), and acridine orange (AO) were purchased from Sigma-Aldrich (St. Louis, MO). ATP, poly(dA:dT), Pam3CSK4, muramyl dipeptide (MDP), FLA-ST, nanoparticles of silicon dioxide (Nano-SiO2), and rapamycin were purchased from InvivoGen (San Diego, CA). CC and MSU were prepared as described previously (16, 17). Antibodies against ASC (cat. No.: sc-22514-R), IL-18 (cat. No.: sc-6177), COX-2 (cat. No.: sc-1747), and actin (cat. No.: sc-47778) were obtained from Santa Cruz Biotechnology (Santa Cruz, CA). Antibodies against NLRP3 (clone: Cryo-1; cat. No.: AG-20B-0006) and mouse caspase-1 (clone: Casper-2; cat. No.: AG-20B-0044-C100) were purchased from Adipogen International (San Diego, CA). Antibody against IL-1β (cat. No.: AB-401-NA) and CXCL1 ELISA kit were purchased from R&D Systems (Minneapolis, MN). Antibodies against LC3B (cat. No.: NB100-2220), ATG5 (cat. No.: M153-3), and p62 (cat. No.: #5114) were purchased from Novus Biologicals (Littleton, CO). Antibodies against cathepsin B (cat. No.: 06-480-I) and Sirt1 (cat. No.: 07-131) were purchased from Millipore (Bedford, MA). Antibodies against IKKβ (clone: D30C6; cat. No.: #8943), p-IKKα/β (clone: 16A6; cat. No.: #2697), and p-IκBα (clone: 14D4; cat. No.: #2859) were purchased from Cell Signaling Technology (Danvers, MA). ELISA kits for IL-1β, IL-6, TNFα, and MCP-1 were purchased from Affymetrix eBioscience (San Diego, CA). ELISA kit for active caspase-1 was purchased from IBL-America (Minneapolis, MN).

### Cell culture

The J774A.1 macrophages were obtained from the American Type Culture Collection (Rockville, MD). For generating protein knockdown J774A.1 macrophages, cells were transfected with CRISPR/Cas9 knockout plasmids targeting LC3 (cat. No.: sc-426563 and sc-417828-HDR, Santa Cruz Biotechnology) or Sirt1 (cat. No.: sc-430046 and sc-430046-HDR, Santa Cruz Biotechnology). The cells were selected by puromycin (InvivoGen) and the expression levels of LC3 and Sirt1 were checked by Western blot. Bone-marrow-derived macrophages (BMDM) were prepared from bone-marrow of C57BL/6 mice femur and tibia by incubating the cells for 7 days in culture medium containing M-CSF (Peprotech, London, UK).

### Cytokine detection

For IL-1β analysis, cells were incubated for 5 h with LPS (1 μg/ml) followed by incubation for 0.5 h with CVL. Cells were then incubated with NLRP3 activator as indicated in the figure legend. For IL-6, TNFα, and NO analysis, cells were incubated for 0.5 h with CVL followed by incubation for 6 h (IL-6 and TNFα) or 24 h (NO) with LPS (1 μg/ml). The levels of IL-1β, IL-6, and TNFα in the supernatants were measured by ELISA, and the levels of NO in the supernatants were measured by Griess reaction.

### Intracellular ROS and mitochondrial integrity analysis

For intracellular ROS analysis, J774A.1 macrophages were incubated for 0.5 h with CVL (20 μM) or NAC (10 mM) followed by incubation for 0–80 min with LPS (1 μg/ml). Intracellular ROS production was measured by staining the cells with 2′,7′-dichlorofluorescein diacetate (2 μM) (Molecular Probes, Eugene, OR) as described previously (18). For mitochondrial integrity and mitochondrial ROS analysis, J774A.1 macrophages were incubated for 5 h with LPS (1 μg/ml) followed by incubation for 0.5 h with CVL. Cells were then incubated with CC (100 μg/ml) for 24 h. The mitochondrial integrity was measured by staining the cells with MitoTracker Deep Red (25 nM) and MitoTracker Green (25 nM) (Thermo Scientific, Rockford, IL). The mitochondrial ROS production was measured by staining the cells with MitoSOX (5 μM) (Thermo Scientific, Rockford, IL). Data were acquired by flow cytometry.

### Cathepsin B activity assay

J774A.1 macrophages incubated for 5 h with LPS (1 μg/ml) followed by incubation for 0.5 h with CVL (20 μM). Cells were then incubated with CC (100 μg/ml) for 24 h. Cathepsin B activity in the cells was measured by the Magic Red Cathepsin B Detection Kit according to the manufacturer's instructions (ImmunoChemistry, #937). The results were visualized using an Olympus FV 1000-IX81 confocal microscope.

### Autophagy analysis

J774A.1 macrophages were incubated for 0–24 h with CVL (20 μM). The levels of LC3, p62, and ATG5 in the cell lysates were measured by Western blot. For MDC and AO staining, J774A.1 macrophages were incubated for 12 h with CVL (20 μM) or 4 h with rapamycin (100 nM). Autophagolysosomes and lysosomes were measured by staining with MDC (50 nM) and AO (1 μg/ml), respectively, and visualized by fluorescence microscopy.

### ASC oligomerization analysis

J774A.1 macrophages were incubated for 5 h with LPS (1 μg/ml) followed by incubation for 0.5 h with CVL (20 μM). Cells were then incubated with CC (100 μg/ml) for 24 h. The procedures for ASC oligomerization analysis were modified from a previous study (19). Cells were lysed in ice-cold lysis buffer (1% Nonidet-P40, 10% glycerol, 50 mM Tris, pH 7.8, 50 mM NaCl, and 5 mM EDTA) and then centrifuged at 330 × g for 10 min at 4°C. The pellets were washed by ice-cold PBS, resuspended in 500 μl of ice-cold PBS and crosslinked with 2 mM disuccinimidylsuberate for 30 min at room temperature. After centrifugation at 330 × g for 10 min at 4°C, the pellets were analyzed by Western blot using an ASC antibody. For ASC speck formation, cells were fluorescently labeled with ASC antibody and visualized by an Olympus BX-41 microscope.

### NF-κB reporter assay

NF-κB reporter cells (J-Blue cells) are derived from J774A.1 macrophages stably transfected with the NF-κB-inducible reporter plasmid (pNiFty2-SEAP, InvivoGen: cat. code: pnifty2-seap). J-Blue cells were seeded in 24-well plates at a density of 2 × 10^5^ cells/ml and were grown overnight at 37°C in a 5% CO_2_ incubator. The cells were pretreated with CVL (5, 10, 20 μM), NAC (10 mM), or DMSO (vehicle) for 30 min and then treated with LPS (1 μg/ml) for 24 h. The supernatants were harvested, and NF-κB transcriptional activity was assayed using QUANTI-Blue^TM^ alkaline phosphatase detection medium (InvivoGen: cat. code: rep-qb1) according to the instruction manual.

### Pyroptosis detection

J774A.1 macrophages were incubated for 5 h with LPS (1 μg/ml) followed by incubation for 0.5 h with CVL (20 μM). Cells were then incubated with ATP (5 mM) for 0.5 h. The cell viability was measured by the AlamarBlue® assay kit according to the instruction manual (AbD Serotec Ltd). The LDH release assay was performed by the CytoTox 96® Non-radioactive Cytotoxicity assay kit according to the instruction manual (Promega, Madison, WI). The membrane integrity was measured by staining the cells with 40 μg/ml of propidium iodide (PI) (Sigma-Aldrich) and analyzed by flow cytometry. The cell size was determined by calculating 20 representative cells using ImageJ software.

### Mouse disease model

Seven-to-nine-week-old male C57BL/6JNal mice were purchased from the National Laboratory Animal Center (Taipei, Taiwan). The mice were housed in a room controlled for temperature (23 ± 3°C) and relative humidity (40–60%). The mice were acclimated in the animal facility for at least a week before the experiments. Animal experiments were performed with the approval of the Institutional Animal Care and Use Committee of the National Ilan University (approval number: 106-13) and in accordance with the NIH Guide for the Care and Use of Laboratory Animals. The mice were randomized into four groups: Group I [control; orally administered vehicle (200 μl) at 0, 24, and 48 h; one intraperitoneal (i.p.) injection with sterile PBS (0.5 ml) at 49 h; *n* = 3], Group II [MSU treatment; orally administered vehicle (200 μl) at 0, 24, and 48 h; one i.p. injection with MSU (3 mg in 0.5 ml of PBS) at 49 h; *n* = 6], Group III [CVL+MSU treatment; orally administered CVL (20 mg/kg) at 0, 24, and 48 h; one i.p. injection with MSU at 49 h; *n* = 6], Group IV [colchicine+MSU treatment, one i.p. injection with colchicine (1 mg/kg) at 48 h, one i.p. injection with MSU at 49 h, *n* = 5] and Group V [CVL treatment; orally administered CVL (20 mg/kg) at 0, 24, and 48 h; one i.p. injection with sterile PBS (0.5 ml) at 49 h; *n* = 3]. The mice were euthanized by cervical dislocation at 53 h, and the peritonea were lavaged with 3 ml of ice-cold PBS. The levels of IL-1β, IL-6, TNFα, MCP-1, active casapse-1, and CXCL1 in the peritoneal lavage fluids were measured by ELISA. The levels of IL-18 and ASC in the peritoneal lavage fluids were measured by Western blot. The levels of NLRP3 and HO-1 in the peritoneal cells were measured by Western blot.

### Statistical analysis

Data are shown as the mean ± SD from at least three independent experiments. All experimental results were analyzed using one-way ANOVA or 2-tailed paired Student's *t*-test. *p* < 0.05 was considered statistically significant.

## Results

### CVL reduced NLRP3 inflammasome activation

**Figure 1:**
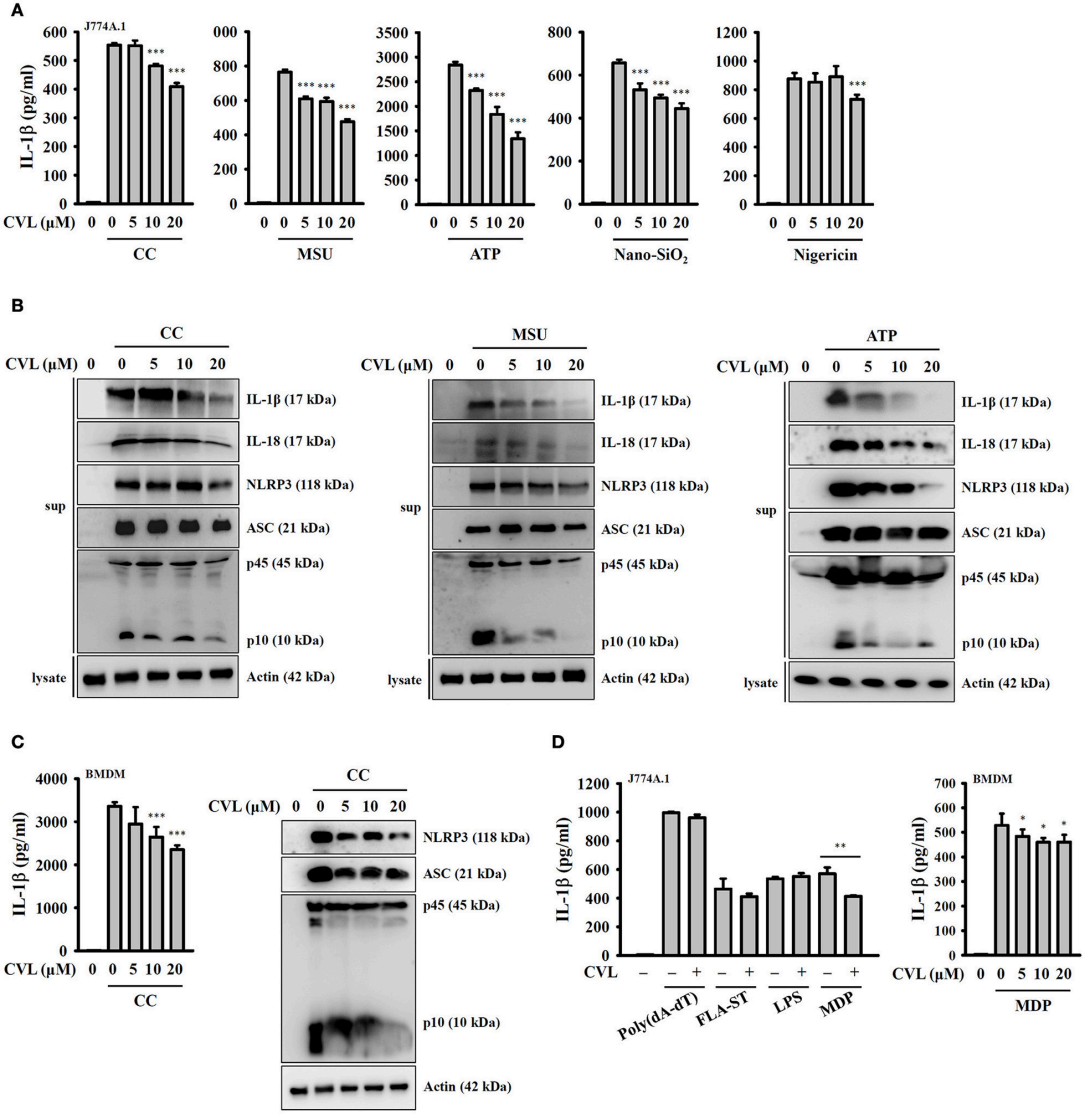
CVL reduced NLRP3 inflammasome activation. **(A)** J774A.1 macrophages were incubated for 5 h with LPS (1 μg/ml) (LPS priming) followed by incubation for 0.5 h with CVL. Cells were then incubated with CC (100 μg/ml, 24 h), MSU (100 μg/ml, 24 h), ATP (5 mM, 0.5 h), nigericin (10 μM, 0.5 h), and nano-SiO_2_ (100 μg/ml, 24 h). **(B)** LPS-primed J774A.1 macrophages were incubated for 0.5 h with CVL followed by incubation with CC (100 μg/ml, 24 h), MSU (100 μg/ml, 24 h), and ATP (5 mM, 0.5 h). **(C)** LPS-primed BMDM were incubated for 0.5 h with CVL followed by incubation with CC (100 μg/ml) for an additional 24 h. **(D)** LPS-primed or Pam3CSK4-primed (for LPS transfection only) cells were incubated for 0.5 h with CVL followed by transfection with poly(dA/dT) (2 μg/ml, 6 h), FLA-ST (1 μg/ml, 6 h), MDP (10 μg/ml, 6 h), or LPS (2 μg/ml, 6 h). The levels of IL-1β, IL-18, NLRP3, ASC, and caspase-1 in the culture medium were measured by Western blot. The IL-1β levels in the supernatants were measured by ELISA. The Western blot results are representative of three different experiments. The ELISA data are expressed as the mean ± SD of three separate experiments. *, **, and *** indicate a significant difference at the level of *p* < 0.05, *p* < 0.01, and *p* < 0.001, respectively, compared to activator-treated cells.

To investigate whether CVL can inhibit the NLRP3 inflammasome, we incubated LPS-primed J774A.1 macrophages with CVL for 30 min before they were stimulated with activators of the NLRP3 inflammasome. The results indicated that CVL significantly reduced the IL-1β secretion induced by CC, MSU, ATP, and Nano-SiO2 but showed lower inhibitory activity in nigericin-stimulated cells (Figure 1A). To further confirm the inhibitory effect of CVL on the NLRP3 inflammasome, we measured the expression levels of IL-1β, IL-18, NLRP3, ASC, and caspase-1 in the supernatants of CC-, MSU-, and ATP-stimulated J774A.1 macrophages by Western blot. The results showed that CVL significantly reduced the expression levels of IL-1β, IL-18, NLRP3, and caspase-1 but only slightly reduced ASC expression (Figure 1B). The inhibitory effect of CVL on CC-mediated secretion of IL-1β, NLRP3, ASC, and active caspase-1 were confirmed in LPS-primed mouse BMDM (Figure 1C). We further investigated whether CVL specifically inhibits the NLRP3-dependent inflammasome. We found that CVL was selective for NLRP3-dependent inflammasome stimuli, as CVL did not reduce IL-1β secretion in J774A.1 macrophages transfected with poly(dA:dT), FLA-ST (flagellin from S. typhimurium), and LPS, which are dependent on the AIM2, NLRC4, and non-canonical inflammasomes, respectively (Figure 1D). Interestingly, CVL slightly reduced IL-1β secretion in both J774A.1 macrophages and BMDM transfected with the NLRP1 inflammasome activator MDP (Figure 1D). These results indicate that CVL selectively inhibited NLRP3 inflammasome activation in macrophages.

### CVL reduced caspase-1-dependent pyroptosis

**Figure 2:**
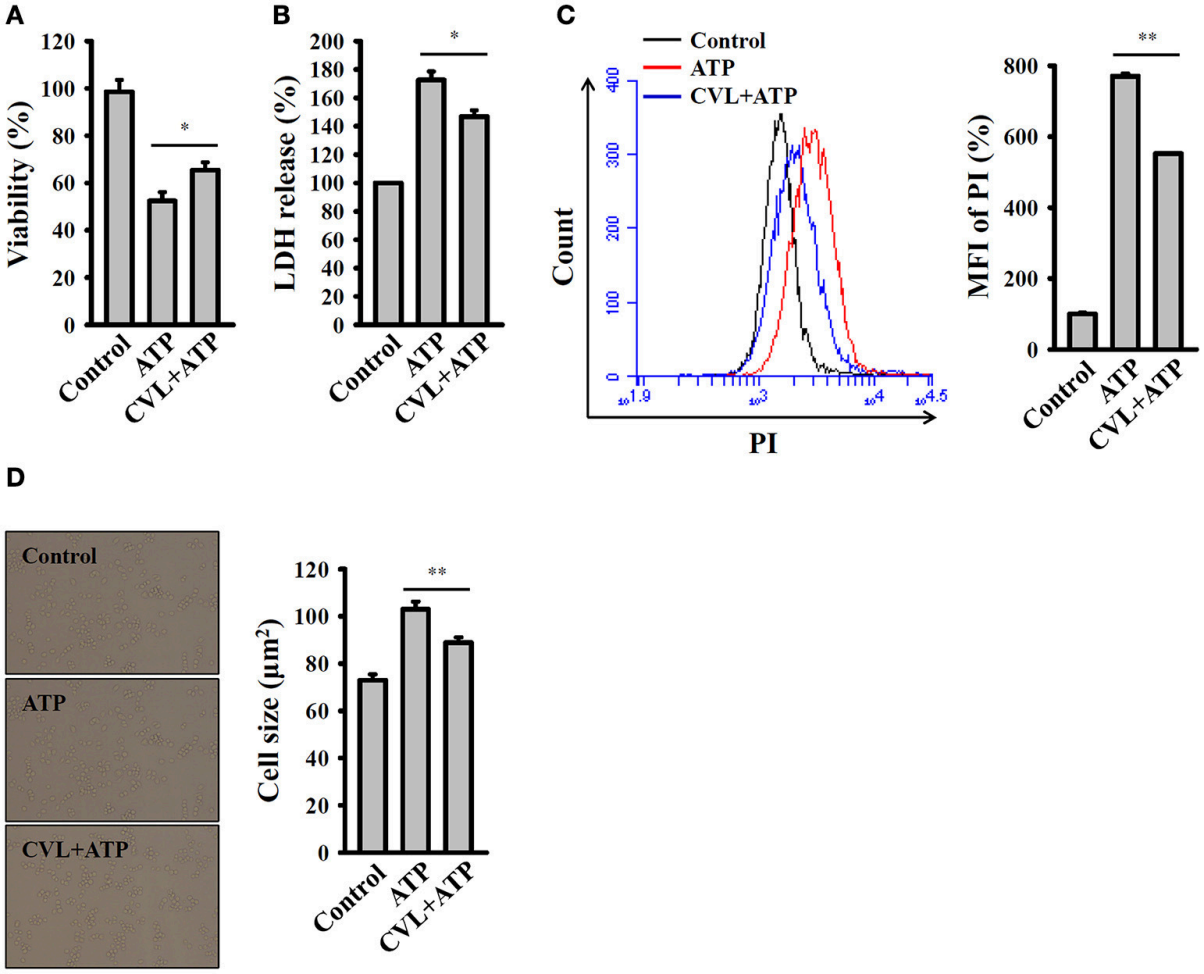
CVL reduced caspase-1-dependent pyroptosis. J774A.1 macrophages were incubated for 5 h with LPS (1 μg/ml) followed by incubation for 0.5 h with CVL (20 μM). Cells were then incubated for 0.5 h with ATP (5 mM). **(A)** Cell viability was assayed by the AlamarBlue cell viability assay kit. **(B)** LDH release was assayed by the CytoTox 96 Non-radioactive Cytotoxicity assay kit. **(C)** The membrane integrity was measured by staining the cells with 40 μg/ml of PI, and analyzed by flow cytometry. **(D)** The cell size was determined by calculating 20 representative cells using ImageJ software. The data are expressed as the means ± SD for three separate experiments. * and ** indicates a significant difference at the level of *P* < 0.05 and *P* < 0.01, respectively.

Pyroptosis is a type of caspase-1-dependent cell death, which is often associated with inflammasome activation and IL-1β production (18). We investigated the effect of CVL on the pyroptosis in mouse macrophages. Pyroptosis is characterized by a loss of cell membrane integrity that leads to fluid influx and cell swelling (18). We found that that cell viability was reduced by ATP in the LPS-primed J774A.1 macrophages, and this effect was reversed by CVL (Figure 2A). To confirm the effect of CVL on the cell death, an LDH release assay was used. We found that the LDH release induced by ATP in the LPS-primed J774A.1 macrophages was significantly reduced by CVL (Figure 2B). In addition, a membrane-impermeant fluorescent dye, PI, uptake assay was used to quantitatively examine membrane integrity (18). We found that that propidium iodide uptake was increased by ATP in the LPS-primed J774A.1 macrophages, and this effect was suppressed by CVL (Figure 2C). These results indicated that CVL preserved membrane integrity in NLRP3 inflammasome-activated cells. Furthermore, to determine whether CVL affects fluid influx and cell swelling, cell size was quantified after NLRP3 inflammasome activation. We found that ATP treatment increased the cell size (102.9 ± 3.2 μm^2^) compared to the control cells (72.9 ± 2.5 μm^2^), and CVL significantly reduced the cell size increase induced by ATP (88.8 ± 2.2 μm^2^) (Figure 2D). These results indicated that CVL reduced caspase-1-dependent pyroptosis.

### CVL inhibition of the NLRP3 inflammasome is independent of the priming signal

**Figure 3:**
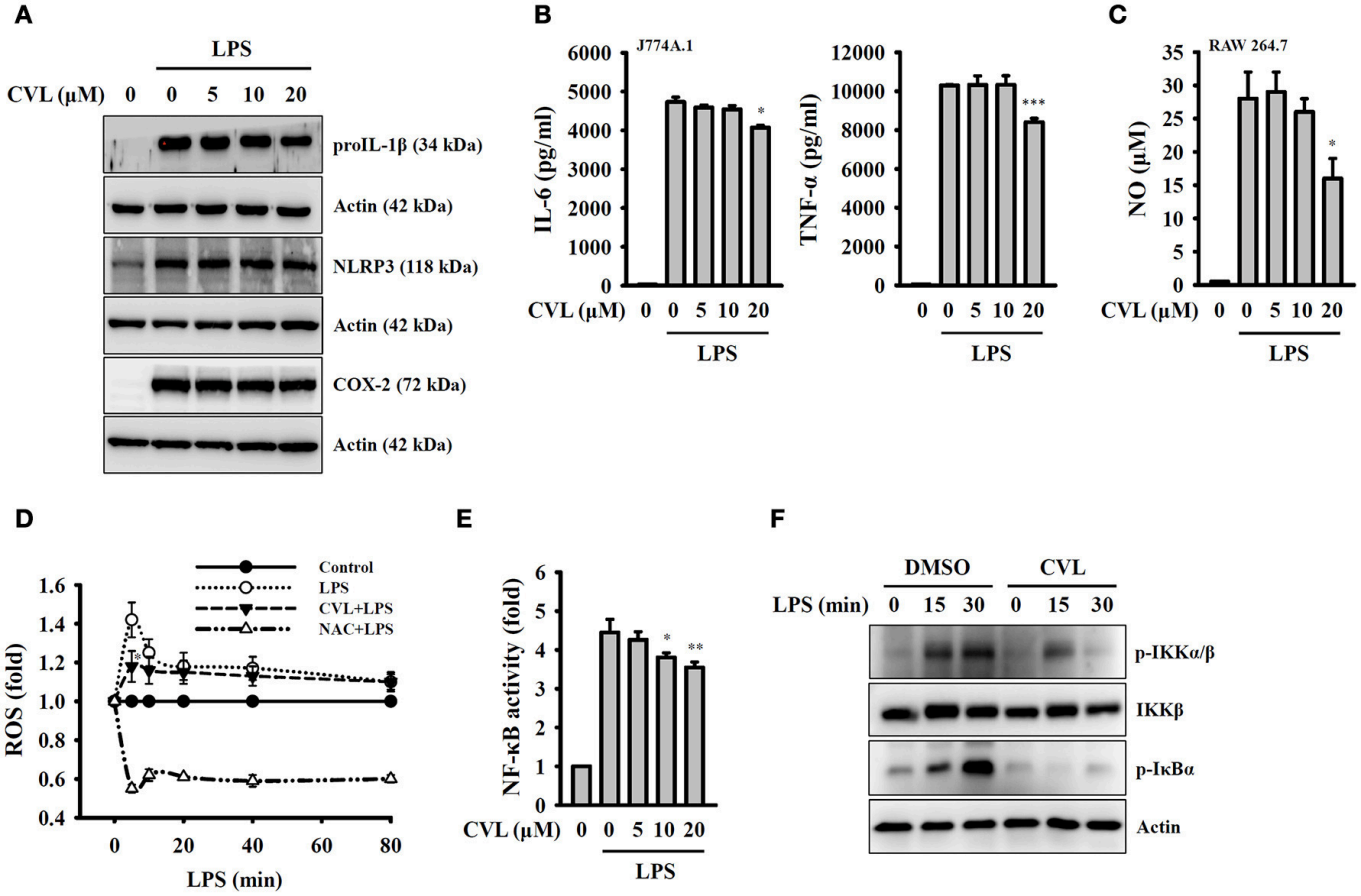
CVL inhibited the NLRP3 inflammasome independent of the priming signal. **(A,B)** J774A.1 macrophages were incubated for 0.5 h with CVL followed by incubation for 6 h with LPS (1 μg/ml). The levels of NLRP3, proIL-1β, and COX-2 in the cell lysates were measured by Western blot **(A)**, and the levels of IL-6 and TNF-α in the culture medium were measured by ELISA **(B)**. **(C)** RAW 264.7 macrophages were incubated for 0.5 h with CVL followed by incubation for 24 h with LPS (1 μg/ml). The NO levels in the culture medium were measured by Griess reagent. **(D)** J774A.1 macrophages were incubated for 0.5 h with CVL (20 μM) or NAC (10 mM) followed by incubation for 0–80 min with LPS (1 μg/ml). The ROS levels in the cells were measured by 2′,7′-dichlorofluorescein diacetate. **(E)** J-Blue cells were incubated for 0.5 h with CVL or NAC (10 mM) followed by incubation for 24 h with LPS (1 μg/ml). The NF-κB activity was assayed using QUANTI-Blue^TM^ alkaline phosphatase detection medium. **(F)** BMDM were incubated for 0.5 h with CVL or DMSO (vehicle) followed by incubation for 0–30 min with LPS (1 μg/ml). The phosphorylation levels of IKKα/β and IκBα in the cell lysates were measured by Western blot. The Western blot results are representative of three different experiments. The ELISA and NF-κB data are expressed as the mean ± SD of three separate experiments. *, ** and *** indicate a significant difference at the level of *p* < 0.05, *p* < 0.01 and *p* < 0.001, respectively, compared to LPS-treated cells.

Full activation of the NLRP3 inflammasome requires both priming and activation signals, the former controlling the expression of NLRP3 and proIL-1β and the latter controlling NLRP3 inflammasome assembly and caspase-1 activation (8). We investigated whether CVL inhibited the NLRP3 inflammasome by affecting the expression of NLRP3 and proIL-1β in LPS-activated macrophages. We found that CVL did not significantly affect the expression levels of NLRP3, proIL-1β, and COX-2 in LPS-activated J774A.1 macrophages (Figure 3A). In addition, CVL slightly reduced the expression levels of IL-6, TNF-α (Figure 3B) and NO (Figure 3C) at a high concentration (20 μM). We further investigated the effect of CVL on LPS-mediated ROS production, which is an important regulator of the priming signal of the NLRP3 inflammasome (20). We found that CVL reduced ROS production only at 5 min after LPS stimulation (*p* < 0.05), but had no significant effect on LPS-induced ROS production between 10 and 80 min (Figure 3D). Furthermore, NF-κB activation also plays important role in LPS-mediated NLRP3 and proIL-1β expression. Here, we demonstrated that CVL significantly inhibited LPS-induced NF-κB activation in J774A.1 macrophages using an NF-κB reporter assay (Figure 3E). The NF-κB inhibitory effect was confirmed in BMDM, as CVL reduced the phosphorylation levels of IKKα/β and IκBα in LPS-activated BMDM (Figure 3F). These results indicated that although CVL inhibited IL-6, TNF-α, and NO production through reducing NF-κB activation, CVL did not inhibit the priming signal of the NLRP3 inflammasome.

### CVL reduced CC-mediated lysosomal rupture, mitochondrial damage, and ASC oligomerization

**Figure 4:**
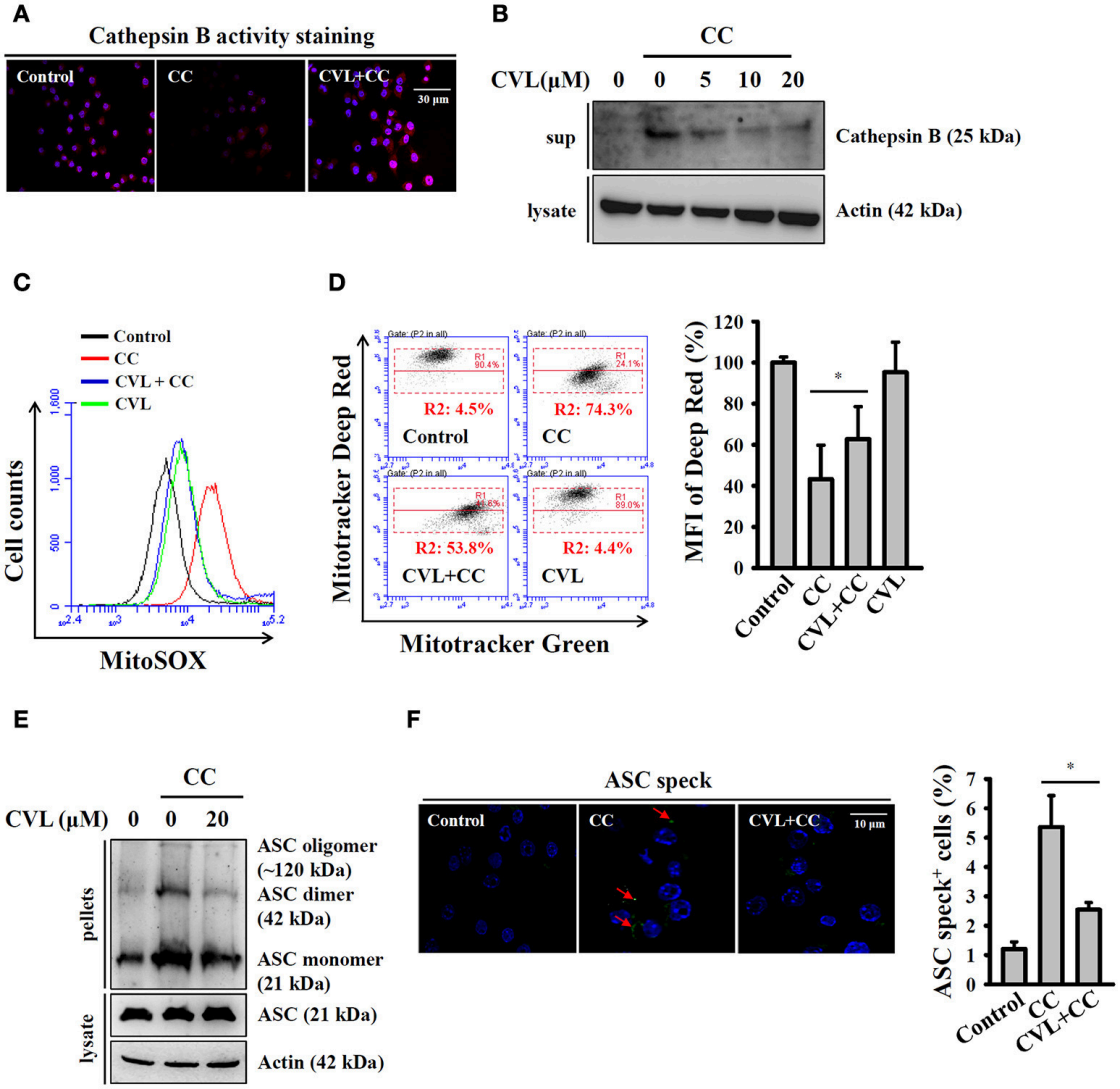
CVL reduced CC-mediated lysosomal rupture, mitochondrial damage and ASC oligomerization. **(A)** LPS-primed J774A.1 macrophages were incubated for 0.5 h with CVL (20 μM) followed by incubation with CC (100 μg/ml) for an additional 24 h. Cathepsin B activity in the cells was measured by a Cathepsin B Activity Kit. **(B)** LPS-primed J774A.1 macrophages were incubated for 0.5 h with CVL followed by incubation with CC (100 μg/ml) for an additional 24 h. The cathepsin B levels in the culture medium were measured by Western blot. **(C,D)** LPS-primed J774A.1 macrophages were incubated for 0.5 h with CVL (20 μM) followed by incubation with CC (100 μg/ml) for an additional 24 h. Mitochondrial ROS were measured by staining with MitoSOX **(C)**, and the mitochondrial integrity was measured by staining with MitoTracker Deep Red and MitoTracker Green **(D)**. The MFI of Mitotracker Deep Red are expressed as the means ± SD for three separate experiments. **(E,F)** LPS-primed J774A.1 macrophages were incubated for 0.5 h with CVL (20 μM) followed by incubation with CC (100 μg/ml) for an additional 24 h. The levels of ASC oligomerization were measured by Western blot after the lysates were crosslinked with disuccinimidylsuberate **(E)**, and ASC speck formation was assayed by fluorescence microscopy **(F)**. The Western blot results are representative of three different experiments. The percentage of ASC speck positive cells are expressed as the means ± SD for three separate experiments. * indicates a significant difference at the level of *P* < 0.05.

Phagocytosis of crystal particles by macrophages allows these particles to physically penetrate lysosome membranes and leads to lysosomal rupture, which results in the release of the cathepsin B into cytosol; this protein binds to NLRP3 and triggers NLRP3 inflammasome activation (16, 21). We asked whether CVL inhibited the CC-mediated NLRP3 inflammasome by reducing lysosomal rupture. As shown in Figure 4A, CC treatment reduced cathepsin B activity (lower red fluorescent signals), which indicated the leakage of cathepsin B into the cytoplasm, where the enzyme activity was significantly reduced by the neutral pH. We found that CVL reversed the cathepsin B activity in CC-treated cells, indicating that lysosomal rupture was reduced (Figure 4A). The protective effect of CVL on the lysosome was confirmed, as the mature form of cathepsin B was detected in the culture medium after CC treatment, and this effect was reduced by CVL (Figure 4B). Cathepsin B released from ruptured lysosomes increases mitochondrial stress and promotes ROS production from the mitochondria (22). Damaged mitochondria play important roles in the NLRP3 inflammasome (23). Thus, we investigated whether CVL inhibited the NLRP3 inflammasome by protecting the mitochondria from damage. We found that CC treatment increased the mitochondrial ROS production, and this effect was reduced by CVL (Figure 4C). In addition, CC treatment resulted in decrease staining of MitoTracker Deep Red, a fluorescent probe that stains mitochondria with intact membranes, indicating that the mitochondria were damaged by CC (Figure 4D). CVL reduced the mitochondrial damage in CC-treated cells, as it increased the MitoTracker Deep Red staining (Figure 4D). Additionally, ASC oligomerization is the crucial step for full activation of the NLRP3 inflammasome (24). We examined whether CVL can disrupt ASC oligomerization in CC-activated J774A.1 macrophages. We found that ASC oligomerization was observed in CC-stimulated cells, and this effect was significantly reduced by CVL (Figure 4E). The effect of CVL on ASC oligomerization was further investigated by measuring ASC speck formation. The numbers of ASC speck-positive cells were increased significantly after CC stimulation, and this effect was significantly reduced by CVL (Figure 4F). These results indicated that CVL inhibited CC-mediated NLRP3 inflammasome activation partially through reduction of lysosomal rupture, mitochondrial damage and ASC oligomerization.

### CVL inhibited the NLRP3 inflammasome by enhancing autophagy

**Figure 5:**
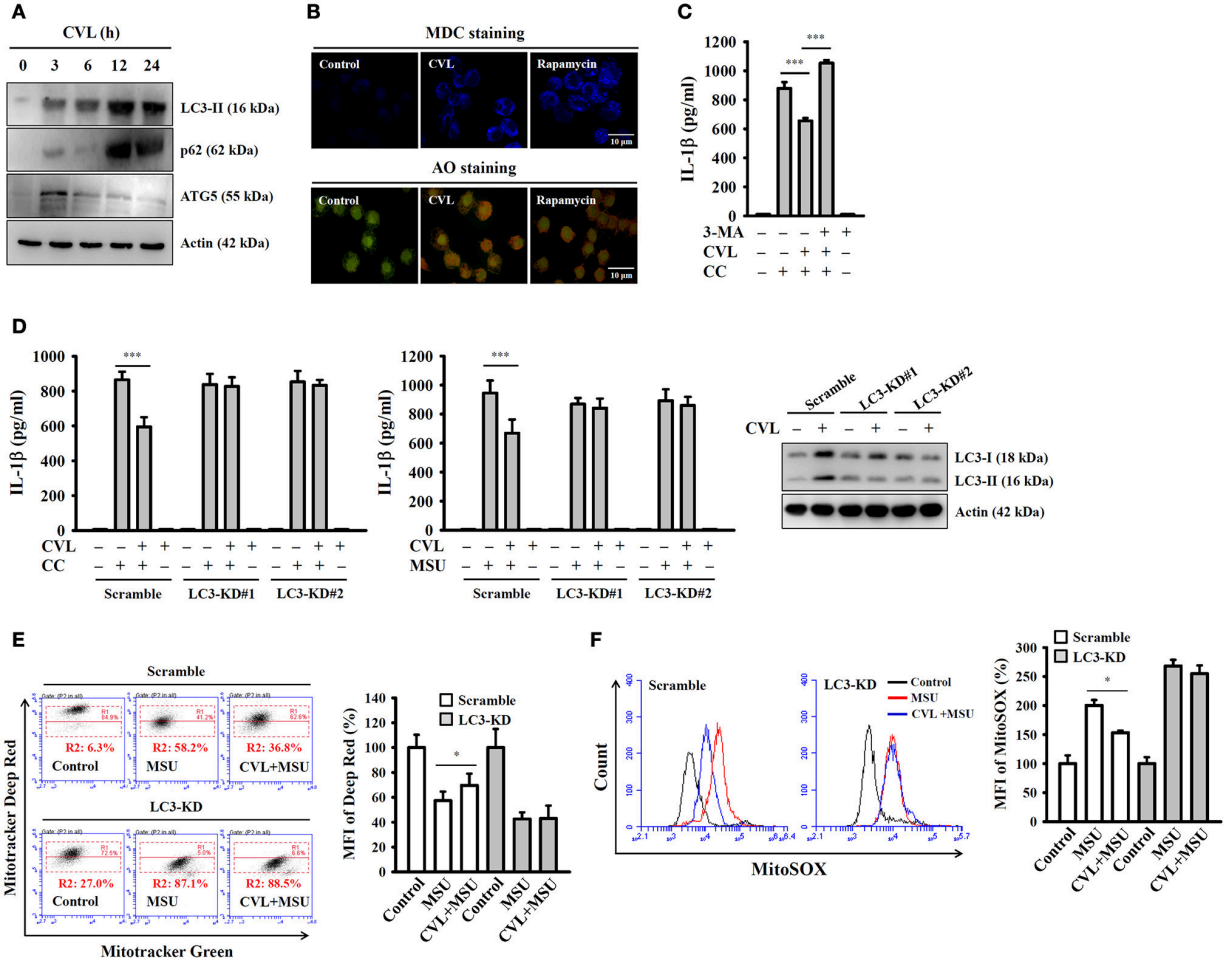
CVL inhibited the NLRP3 inflammasome by enhancing autophagy. **(A)** J774A.1 macrophages were incubated for 0–24 h with CVL (20 μM). The levels of LC3, p62, and ATG5 in the cell lysates were measured by Western blot. **(B)** J774A.1 macrophages were incubated with CVL (20 μM, 12 h) or rapamycin (100 nM, 4 h). Accumulation of the fluorescent signals of MDC and AO was measured by confocal microscopy. **(C)** LPS-primed J774A.1 macrophages were incubated for 0.5 h with 3-MA (5 mM) and CVL (20 μM) followed by incubation with CC (100 μg/ml) for an additional 24 h. The levels of IL-1β in the culture medium were measured by ELISA. **(D)** LPS-primed wild-type (scramble) and LC3-knockdown J774A.1 macrophages (clones #1 and #2) were incubated for 0.5 h with CVL (20 μM) followed by incubation with CC (100 μg/ml) or MSU (100 μg/ml) for an additional 24 h. The levels of IL-1β in the culture medium were measured by ELISA. The expression levels of LC3 in the wild-type and LC3-knockdown J774A.1 macrophages were measured by Western blot. **(E,F)** LPS-primed wild-type and LC3-knockdown J774A.1 macrophages were incubated for 0.5 h with CVL (20 μM) followed by incubation with MSU (100 μg/ml) for an additional 24 h. Mitochondrial integrity was measured by staining with MitoTracker Deep Red and MitoTracker Green **(E)**, and mitochondrial ROS was measured by staining with MitoSOX **(F)**. The MFI of Mitotracker Deep Red are expressed as the means ± SD for three separate experiments. The Western blot results are representative of three different experiments. The ELISA data are expressed as the mean ± SD of three separate experiments. * and *** indicate a significant difference at the level of *p* < 0.05 and *p* < 0.001, respectively.

Autophagy is a self-protective cellular process that facilitates the turnover of damaged proteins and organelles. Accumulating evidence has indicated that autophagy negatively regulates NLRP3 inflammasome activation (25, 26). These findings prompted us to investigate whether CVL inhibited the NLRP3 inflammasome through autophagic induction. We found that the expression levels of the autophagy markers LC3-II and p62 were increased by CVL, and the level reached its maximum at 12 h; it then began to decline at 24 h and remained higher than that of control cells (Figure 5A). In addition, incubation with CVL for 3 h induced another autophagy marker ATG5 expression; however, it began to decline after 6 h (Figure 5A). The autophagic induction activity of CVL was further confirmed by staining cells with MDC, a specific autophagolysosome marker, and AO, a cell-permeable fluorescent dye that stains acidic organelles present in increased numbers during autophagy (18). We found that accumulation of the fluorescent signals of MDC and AO was higher in CVL-treated J774A.1 macrophages than in control cells (Figure 5B). These results indicated that CVL induced autophagy in macrophages. We further asked whether autophagy is responsible for CVL-mediated NLRP3 inflammasome inhibition. We found that CVL significantly inhibited CC-induced IL-1β secretion, and this effect was reversed by the autophagy inhibitor 3-MA (Figure 5C). The role of autophagy in CVL-mediated inhibition of the NLRP3 inflammasome was further confirmed by CRISPR/Cas9-mediated LC3-knockdown technology in J774A.1 macrophages, as CVL failed to inhibit CC- and MSU-induced IL-1β secretion in LC3-knockdown cells (Figure 5D). Additionally, CVL protected mitochondria from damage (Figure 5E) and reduced mitochondrial ROS production (Figure 5F) in MSU-activated macrophages, and this effect was impaired in LC3-knockdown cells. These results indicated that CVL inhibited the NLRP3 inflammasome by enhancing autophagy.

### CVL inhibited the NLRP3 inflammasome by enhancing the Sirt1/autophagy axis

**Figure 6:**
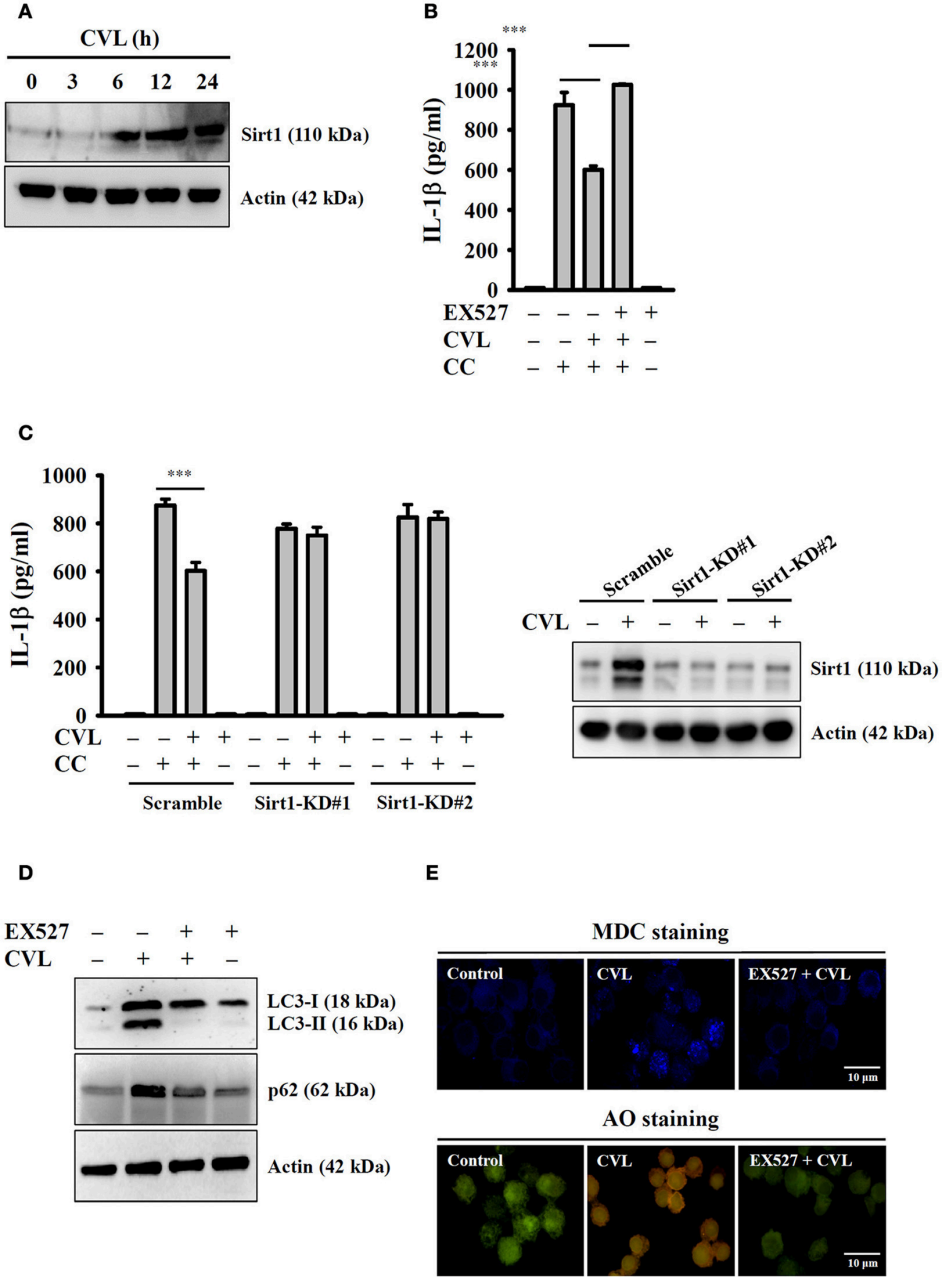
CVL inhibited the NLRP3 inflammasome by enhancing the Sirt1/autophagy axis. **(A)** J774A.1 macrophages were incubated for 0–24 h with CVL (20 μM). The Sirt1 levels in the cell lysates were measured by Western blot. **(B)** LPS-primed J774A.1 macrophages were incubated for 0.5 h with EX527 (10 μM) and CVL (20 μM) followed by incubation with CC (100 μg/ml) for an additional 24 h. The IL-1β levels in the culture medium were measured by ELISA. **(C)** LPS-primed wild-type (scramble) and Sirt1-knockdown J774A.1 macrophages (clones #1 and #2) were incubated for 0.5 h with CVL (20 μM) followed by incubation with CC (100 μg/ml) for an additional 24 h. The IL-1β levels in the culture medium were measured by ELISA. The expression levels of Sirt1 in the wild-type and Sirt1-knockdown J774A.1 macrophages were measured by Western blot. **(D,E)** J774A.1 macrophages were incubated for 0.5 h with EX527 (10 μM) followed by incubation with CVL (20 μM) for an additional 12 h. The levels of LC3 and p62 in the cell lysates were measured by Western blot **(D)**, and the accumulation of the fluorescent signals of MDC and AO was measured by confocal microscopy **(E)**. The Western blot results are representative of three different experiments. The ELISA data are expressed as the mean ± SD of three separate experiments. *** indicates a significant difference at the level of *p* < 0.001.

Recent studies showed that Sirt1 inhibited the NLRP3 inflammasome, and this effect was involved in the regulation of autophagic induction (27). We next aimed to determine the effect of CVL on Sirt1 expression and whether Sirt1 can regulate the NLRP3 inflammasome through autophagy. We found that CVL induced Sirt1 expression (Figure 6A), and inhibition of Sirt1 by EX527 promoted CC-mediated IL-1β secretion in CVL-treated J774A.1 macrophages (Figure 6B). Additionally, CVL could not inhibit CC-mediated IL-1β secretion in Sirt1-knockdown J774A.1 macrophages (Figure 6C), which confirmed the role of Sirt1 in CVL-mediated IL-1β down-regulation. Notably, the Sirt1 inhibitor EX527 reduced the expression levels of LC3-II and p62 (Figure 6D) as well as the MDC and AO staining in CVL-treated cells (Figure 6E). These results indicated that CVL inhibited the NLRP3 inflammasome by enhancing the Sirt1/autophagy axis.

### CVL reduced inflammation in a mouse model of MSU-mediated peritonitis

**Figure 7:**
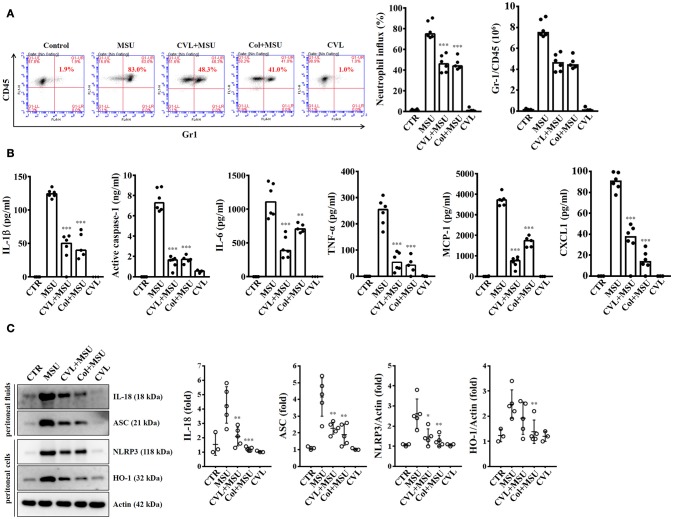
CVL reduced inflammation in a mouse model of MSU-mediated peritonitis. **(A)** Neutrophil influx was quantified by Gr-1 and CD45 staining. **(B)** The levels of IL-1β, active caspase-1, IL-6, TNF-α, MCP-1, and CXCL1 in the peritoneal lavage fluids were measured by ELISA. **(C)** The levels of IL-18 and ASC in the peritoneal lavage fluids and NLRP3 and HO-1 in the peritoneal cells were measured by Western blot. The Western blot results are representative of different experiments and the histogram shows the quantification expressed as the mean ± SD. The ELISA data are expressed as the mean ± SD of three separate experiments. *, **, and *** indicate a significant difference at the level of *p* < 0.05, *p* < 0.01, and *p* < 0.001, respectively, compared to MSU-injected mice. Control group: *n* = 3; MSU group: *n* = 6; CVL+MSU group: *n* = 6; CVL group: *n* = 3.

As CVL inhibited the NLRP3 inflammasome in macrophages, we further investigated the *in vivo* effect of CVL using a mouse model of MSU-mediated peritonitis, which is associated with the NLRP3 inflammasome (17). As shown in Figure 7A, significant peritoneal recruitment of neutrophils was observed in mice injected with MSU. Importantly, when mice received oral gavage of CVL or intraperitoneal injection of colchicine, MSU-induced peritoneal recruitment of neutrophils was strongly impaired (Figure 7A). CVL not only reduced the peritoneal recruitment of neutrophils but also decreased the levels of IL-1β, active caspase-1, IL-6, TNF-α, MCP-1, and CXCL1 in the peritoneal fluids (Figure 7B). CVL also reduced the levels of IL-18 and ASC in the peritoneal fluids and the levels of NLRP3 and HO-1 in the peritoneal cells of MSU-injected mice (Figure 7C). These results indicated that CVL showed anti-NLRP3 inflammasome and anti-inflammatory activity *in vivo*.

## Discussion

Inflammation is a protective immune response triggered by the innate immune system in response to harmful stimuli, such as pathogens, injury and metabolic stress, and its ultimate function is to restore the physiological homeostatic state. These innate immune functions rely on the recognition of pathogen-associated molecular patterns (PAMPs) and danger-associated molecular patterns (DAMPs) by pattern-recognition receptors (PRRs) (28). Inflammasomes are protein complexes expressed in several cell types that assemble in the cytosol after recognition of PAMPs or DAMPs and produce IL-1β and IL-18 (8). IL-1β and IL-18 are two well-documented pro-inflammatory cytokines that play a central role in elevated inflammasome activation. In particular, IL-1β is a potent, multifunctional cytokine that is involved in a host of immune and pro-inflammatory responses (29). Consequently, IL-1β can mediate significant cellular and tissue damage when its expression is up-regulated, as observed in the pathogenesis of acute or chronic inflammatory diseases (29). In this study, our results showed that CVL potently suppressed IL-1β and IL-18 secretion in NLRP3 inflammasome-activated macrophages and mice (Figures 1, 7). These results suggested the possibility of using CVL as a NLRP3 inhibitor for treating NLRP3-associated inflammatory diseases.

Chronic sterile inflammation in the blood vessels plays a major role in the pathogenesis of atherosclerosis; therefore, anti-inflammatory therapy is one potential approach for cardiovascular disease (30). CC and macrophages were shown to appear in early atherosclerotic lesions simultaneously, and uptake of CC by macrophages activates the NLRP3 inflammasome by inducing lysosomal damage (16). Mice lacking NLRP3 inflammasome-related genes, e.g., NLRP3, ASC, and IL-1β, had markedly decreased atherosclerosis compared to wild-type mice in response to a high-cholesterol diet, indicating the important roles of the NLRP3 inflammasome in atherosclerosis (16). Increasing evidence has shown that the NLRP3 inflammasome is a potential drug target for ameliorating atherosclerosis (31). In this study, we found that CVL significantly inhibited CC-induced NLRP3 inflammasome activation by reducing lysosomal and mitochondrial damage (Figure 4). Additionally, gout, a metabolic disease associated with hyperuricaemia and systemic inflammation, is known to be associated with an increased risk of cardiovascular disease (32). NLRP3 inflammasome activation in macrophages stimulated by uric acid crystals increased their lipid accumulation and foam cell formation (33). We found that CVL also significantly suppressed uric acid crystal-induced NLRP3 inflammasome activation in macrophages and in mice (Figures 1, 7). These results indicated that CVL potentially reduced the risk of cardiovascular disease by suppressing the NLRP3 inflammasome activation induced by not only traditional atherogenic factors but also metabolic danger signals.

Although inhibition of inflammasomes prevents pathogenic effects induced by excess inflammation, activation of inflammasomes is required for host defense against pathogen infections (34). Thus, general and non-selective inhibition of inflammasomes may increase the host susceptibility to infection. In this study, we found that CVL specifically inhibited the NLRP3 inflammasome, but it did not significantly affect IL-1β secretion induced by the NLRP1, NLRC4, AIM2, and non-canonical inflammasomes (Figure 1D). The NLRP3 inflammasome not only senses and reacts to infectious pathogens but also responds to metabolic danger signals, e.g., CC, MSU, and saturated fatty acids (7). Inhibition of the NLRP3 inflammasome by CVL may reduce the pathogenic effects induced by the metabolic danger signals, and the side effects of NLRP3 inflammasome inhibition (e.g., increased susceptibility to infection) can be compensated for by other inflammasomes (34).

Full activation of the NLRP3 inflammasome required a pro-inflammatory priming signal that induces NLRP3 and proIL-1β expression (35). In this study, we found that CVL did not affect the priming signal of the NLRP3 inflammasome as evidenced by the unaltered expression of NLRP3 and proIL-1β in LPS-activated J774A.1 macrophages (Figure 3A). Several signaling molecules are involved in regulating the priming signal of the NLRP3 inflammasome, including ROS (20), NF-κB (35), and mitogen-activated protein kinases (36). Although the anti-inflammatory effects of CVL may be associated with its anti-oxidative activity (6), we found that CVL did not significantly inhibit ROS production in LPS-activated macrophages (Figure 3D). Our report and previous studies showed that CVL inhibited NF-κB activation in T cells and endothelial cells (2, 37); however, CVL did not affect NF-κB activation in LPS-activated human monocytes (38). In this study, we demonstrated that CVL inhibited NF-κB activation in LPS-activated J774A.1 macrophages and BMDM (Figures 3E,F). Additionally, although our results showed that CVL did not affect the priming signal of the NLRP3 inflammasome, CVL inhibited production of inflammasome-independent inflammatory mediators, including IL-6, TNF-α, and NO in LPS-activated macrophages (Figures 3B,C). Our results were consistent with those of previous reports showing that CVL inhibited TNF-α expression in LPS-activated human monocytes (38) and in a rat model of periodontitis (39) and also inhibited NO expression in LPS-activated mouse macrophages (40).

Autophagy is emerging as a central process that regulates multiple inflammasome responses at several levels. Although autophagy was initially shown to enhance cell survival, recent reports have observed an inverse relationship between autophagic induction and activation of the NLRP3 inflammasome in macrophages (26). Autophagy regulates the NLRP3 inflammasome through various mechanisms, including direct inhibition of NLRP3 inflammasome activation by removing sources of endogenous NLRP3 agonists, such as damaged mitochondria; suppression of IL-1β secretion by targeting proIL-1β for lysosomal degradation; and selective degradation of inflammasome components, such as NLRP3 and ASC (8, 25). In this study, we demonstrated that CVL acted as a novel autophagy inducer in macrophages (Figure 5). Furthermore, CVL inhibited the NLRP3 inflammasome by reducing mitochondrial damage (Figure 4); notably, these effects were reversed by an autophagy inhibitor and impaired in LC3 knockdown macrophages (Figure 5). Additionally, Sirt1 is one of seven mammalian sirtuins, which comprise a conserved family of NAD-dependent deacetylases and ADP-ribosyltransferases. Sirt1 plays an important role in cell protection against various inflammatory stress conditions (41). Previous studies have indicated that Sirt1 negatively regulated NLRP3 inflammasome activation in mesenchymal stem cells (42) and in vascular endothelial cells (43). Consistent with these observations, our results showed that Sirt1 expression was time-dependently elevated by CVL treatment (Figure 6A), and inhibition of Sirt1 reversed CVL-mediated NLRP3 inflammasome inhibition (Figures 6B,C). In addition, Sirt1 inhibition diminished the autophagic induction of CVL (Figures 6D,E), indicating the important role of Sirt1 in autophagic induction. However, further studies are needed to address the molecular mechanisms underlying CVL upregulation of the Sirt1/autophagy signaling pathway.

The drug CVL is a lipophilic compound with an apparent partition coefficient (log D octanol/H2O) of 3.4 (44). Additionally, CVL is a known membrane “fluidizer,” which alters membrane structure and protein-lipid interactions (45). The known NLRP3 inhibitors, including Bay11-7082, MNS, and acrylamide derivatives, have been suggested to act through direct alkylation of NLRP3 (12). Although such covalent binding has not been observed with direct experimental evidence, such as crystal structures and mass spectrometry, cysteine residues believed to be their alkylating target sites have been examined through computational and modeling analysis (12). Cysteine is frequently involved in the modulation of protein activity and signaling events through the potent chemical reactivity at its thiol group (46). In particular, the cysteine thiol group is easily alkylated (referred to as S-alkylation) by some electrophilic chemicals or peptides, whereas this alkylation can be experimentally inhibited by L-cysteine (47). A previous report showed that CVL also has an electrophilic site that can directly bind to the cysteine at position 166 (48). In addition, previous studies reported that CVL is a pore-blocking drug, and there is an interaction between CVL and the pore located at the cytosolic portion of the inner helix (bundle-crossing region) containing cysteine-166, which is required (49). The replacement of cysteine-166 with alanine completely abolished the pore-blocking effect of CVL (49). In the present study, CVL attenuated the NLRP3 inflammasome, which might be due to NLRP3 alkylation, similar to other known NLRP3 alkylators. These previous observations together with the present results indicate that CVL induces direct alkylation of NLRP3, thereby reducing the elevated activation of the NLRP3 inflammasome, although the exact alkylation site in NLRP3 remains to be identified.

In conclusion, we demonstrated that CVL inhibited the NLRP3 inflammasome by suppressing the activation signals, rather than the priming signals, of the NLRP3 inflammasome in macrophages. The underlying mechanisms for the anti-NLRP3 inflammasome activity were shown to be preserving lysosomal and mitochondrial integrity and augmenting Sirt1/autophagy. Oral administration of CVL also attenuated inflammatory responses in a mouse model of NLRP3-associated peritonitis. These results indicated that CVL has the potential to be repositioned as a novel autophagy inducer for treatment of NLRP3 inflammasome-associated inflammatory diseases.

## Author contributions

K-FH and AC designed the experiments and analyzed data. K-FH, YR, and AC wrote the manuscript. W-TW, L-HL, W-YL, and C-CC performed experiments. S-PY and S-MC contributed to critical revision of the manuscript.

### Conflict of interest statement

The authors declare that the research was conducted in the absence of any commercial or financial relationships that could be construed as a potential conflict of interest.
